# Supplementary material for: Metabolic rewiring in skin epidermis drives tolerance to oncogenic mutations
Source: Nat Cell Biol. 2025 Jan 6;27(2):218–31. doi: 10.1038/s41556-024-01574-w (PMC11821535; doi:10.1038/s41556-024-01574-w)
Supplement: Supplementary file 1 — Reporting Summary [file 41556_2024_1574_MOESM1_ESM.pdf]

nature portfolio

Corresponding author(s): VALENTINA GRECO
RACHEL PERRY

Last updated by author(s): 9/27/2024

Reporting Summary

Nature Portfolio wishes to improve the reproducibility of the work that we publish. This form provides structure for consistency and transparency in reporting. For further information on Nature Portfolio policies, see our Editorial Policies and the Editorial Policy Checklist.

Statistics

For all statistical analyses, confirm that the following items are present in the figure legend, table legend, main text, or Methods section.

n/a	Confirmed	
☐	☒	The exact sample size (*n*) for each experimental group/condition, given as a discrete number and unit of measurement
☐	☒	A statement on whether measurements were taken from distinct samples or whether the same sample was measured repeatedly
☐	☒	The statistical test(s) used AND whether they are one- or two-sided<br>*Only common tests should be described solely by name; describe more complex techniques in the Methods section.*
☒	☐	A description of all covariates tested
☒	☐	A description of any assumptions or corrections, such as tests of normality and adjustment for multiple comparisons
☐	☒	A full description of the statistical parameters including central tendency (e.g. means) or other basic estimates (e.g. regression coefficient) AND variation (e.g. standard deviation) or associated estimates of uncertainty (e.g. confidence intervals)
☐	☒	For null hypothesis testing, the test statistic (e.g. *F*, *t*, *r*) with confidence intervals, effect sizes, degrees of freedom and *P* value noted<br>*Give P values as exact values whenever suitable.*
☒	☐	For Bayesian analysis, information on the choice of priors and Markov chain Monte Carlo settings
☒	☐	For hierarchical and complex designs, identification of the appropriate level for tests and full reporting of outcomes
☒	☐	Estimates of effect sizes (e.g. Cohen's *d*, Pearson's *r*), indicating how they were calculated

Our web collection on statistics for biologists contains articles on many of the points above.

Software and code

Policy information about availability of computer code

Data collection	Inspector PRO, Agilent MSD Chemstation software, Sciex TripleTOF 6600
Data analysis	Image J, MATLAB R2021a, IMARISx64 9.7.2, GraphPad Prism10.3.1, Microsoft Excel Version 2005, MetaboAnalyst 5.0, EL MAVEN(Elucidata.io)

For manuscripts utilizing custom algorithms or software that are central to the research but not yet described in published literature, software must be made available to editors and reviewers. We strongly encourage code deposition in a community repository (e.g. GitHub). See the Nature Portfolio guidelines for submitting code & software for further information.

Data

Policy information about availability of data

All manuscripts must include a data availability statement. This statement should provide the following information, where applicable:

- Accession codes, unique identifiers, or web links for publicly available datasets
- A description of any restrictions on data availability
- For clinical datasets or third party data, please ensure that the statement adheres to our policy

Data Availability statement has been added. " Source data are provided with this study. All other data supporting the findings of this study are available from the corresponding author on reasonable request."

Field-specific reporting

Please select the one below that is the best fit for your research. If you are not sure, read the appropriate sections before making your selection.

☒ Life sciences ☐ Behavioural & social sciences ☐ Ecological, evolutionary & environmental sciences

For a reference copy of the document with all sections, see nature.com/documents/nr-reporting-summary-flat.pdf

Life sciences study design

All studies must disclose on these points even when the disclosure is negative.

Sample size	Three mice were used in general for imaging studies. Exact numbers are detailed in figure legends. Around 5-8 mice were used for mass spec studies- exact n is shown in the graph itself. Each point is plotted from a single animal (described in legends). The sample size was chosen according to previously published papers. For non-imaging experiments also, a minimum of 3 mice was used , again based on previously publications. A sentence has been added to the methods section with references for both imaging and mass spec studies which used similar sample sizes: " No statistical methods were used to pre-determine sample sizes but our sample sizes are similar to those reported in previous publications 56,65,70–74"
Data exclusions	No data was excluded to analyse. Image quantification is described in methods. "No animals were excluded except when measurements could not be completed on a mouse due to variations in laser power or other variations in imaging conditions during a revisit. "- in Methods
Replication	For all imaging experiments, three separate mice imaged at different times (independent biological replicates) are shown. For all non- imaging experiments , a minimum of 3 mice and as many as 7-10 mice (independent biological replicates) were used for mass spectrometry studies in the paper and reported in legends of each experiment with exact n. All attempts at replication were successful and plotted in the manuscript.
Randomization	Mice were first genotyped and identified as control or experimental animals. Within each experimental and control group, animals were chosen randomly for all studies.
Blinding	No blinding was done since the animals are readily identifiable by their toe biopsies and expression of reporter genes during experimentation and analysis. The animals under study need to be clearly identified by their genotype using toe biopsies before data collection.

Reporting for specific materials, systems and methods

We require information from authors about some types of materials, experimental systems and methods used in many studies. Here, indicate whether each material, system or method listed is relevant to your study. If you are not sure if a list item applies to your research, read the appropriate section before selecting a response.

Materials & experimental systems

n/a	Involved in the study	
☐	☒	Antibodies
☐	☒	Eukaryotic cell lines
☒	☐	Palaeontology and archaeology
☐	☒	Animals and other organisms
☒	☐	Human research participants
☒	☐	Clinical data
☒	☐	Dual use research of concern

Methods

n/a	Involved in the study	
☒	☐	ChIP-seq
☒	☐	Flow cytometry
☒	☐	MRI-based neuroimaging

Antibodies

Antibodies used	mouse anti-β-catenin (1:100; BD 610153), rabbit anti-phosphohistoneH3 (Ser10;1:300; Millipore 06-570), donkey anti-mouse AF488 (1:300; ThermoFisher A-21202), goat anti-rabbit AF633 (1:300; ThermoFisher A-21071).
Validation	mouse anti-β-catenin- BD Biosciences has validated specific binding (see technical date sheet of BD 610153). Cited previously in doi: 10.1126/science.1248373.<br>Rabbit anti- phosphohistone H3: Specific binding shown in technical data sheet for Millipore 06-570 anti-phospho histone H3. Cited previously in doi: 10.1038/s41556-021-00670-5

Eukaryotic cell lines

Policy information about cell lines

Cell line source(s)	293T cells were purchased from Thermofisher.

Authentication	The 293T cell line, originally referred as 293tsA1609neo, is a highly transfectable derivative of human embryonic kidney 293 cells, and contains the SV40 T-antigen. It has been authenticated using STR profiling, morphology and expression of SV40 T-antigen(PCR) by ATCC as described in the product page of ATCC and cited widely . Examples: PubMed: 3031469, PubMed: 7690960.
Mycoplasma contamination	No mycoplasma contamination was observed.
Commonly misidentified lines (See ICLAC register)	No commonly misidentified cell line was used in this study.

Animals and other organisms

Policy information about studies involving animals; ARRIVE guidelines recommended for reporting animal research

Laboratory animals	Mouse : Strains used: K14-CreER , Rosa26-CAG-LSL-H2B-mCherry (Jackson Laboratory) R26p-Fucci2 mice were obtained from S. Aizawa (RIKEN), β-cateninflox(Ex3) mice were obtained from M.M. Taketo (Kyoto University) and HrasG12V mice were obtained from S. Beronja (Fred Hutch). Mice of age post P21 were used : For most of the imaging and mass spec studies P45-P65 mice were used. For long term tracking mice of age 3 -4 months were used. Mice of both sex were randomly slected. All animal procedures were approved by the Yale University Institutional Animal Care and Use Committee and housed and fed according to approved protocol 11303. The water and chow (2018SC Rodent diet from Inotiv, US) was autoclaved before use. The housing used is consistent with the Guide for the Care and Use of Laboratory Animals and compliant with the Animal Welfare Act and Regulations. Mice were housed on ventilated Tecniplast lixit racks with ambient temperature of 22°C and 50% ± 10% humidity with a 12 h:12 h light:dark cycle (light on 07:00am -7:00pm) and fed adlib. There are no tumors expected to appear in the experimental time window.
Wild animals	No wild animals were used in this study
Field-collected samples	No field collected samples were used in this study.
Ethics oversight	This study complies with all relevant ethical regulations and all animal procedures were approved by the Yale University Institutional Animal Care and Use Committee under the protocol number 11303 and 20290.

Note that full information on the approval of the study protocol must also be provided in the manuscript.
